# Associations between 44 nonmetric permanent dental traits or anomalies with skeletal sagittal malocclusions and sex, besides correlations across the variations or abnormalities

**DOI:** 10.1186/s12903-022-02481-y

**Published:** 2022-11-26

**Authors:** Negin Ashoori, Fataneh Ghorbanyjavadpour, Vahid Rakhshan

**Affiliations:** 1 Orthodontics Department, School of Dentistry, Ahvaz Jundishapur University of Medical Sciences, Ahvaz, Iran; 2 Department of Dental Anatomy, Dental School, Azad University of Medical Sciences, Tehran, Iran

**Keywords:** Dental anatomy, Nonmetric dental traits, Shape anomalies, Number anomalies, Sex dimorphism, Skeletal malocclusions

## Abstract

**Introduction:**

Nonmetric dental traits and the shape, size, or number of dental anomalies are essential to various dental fields such as orthodontics, dental anatomy, anthropology, pathology, and forensic dentistry. Nonetheless, many are not well assessed worldwide. Moreover, most studies are limited to a few nonmetric traits. Therefore, we aimed to examine several nonmetric dental traits/anomalies.

**Methods:**

In this cross-sectional epidemiological study, ~ 9000 permanent teeth of 331 non-syndromic orthodontic patients (radiographs and dental casts) with fully erupted permanent dentitions (except the third molars and some cases of a few teeth missing or excluded) were evaluated by two observers, each twice, in search for 62 nonmetric traits/shape-number-size anomalies. The traits/anomalies of interest were supernumerary, microdontia, peg-shaped lateral, shovelings, talon cusps, Carabelli cusps, fifth/sixth/seventh cusps on the molars, hypocone/hypoconulid absence, protostylid, deflecting wrinkle‏s, canine mesial ridge, distal trigonid crest, canine distal accessory ridge, accessory cusps in the mesial/distal marginal ridges, mesial/distal accessory ridges, and accessory cusps in the lingual of the mandibular premolars and second molars). Data, at both patient/quarter levels, were analyzed regarding the associated factors (skeletal Angle classes, crowding, sex, and sides) as well as the correlations among traits, using the chi-square test and Spearman correlation coefficient (α = 0.05).

**Results:**

Prevalence rates of 44 traits/anomalies were reported (18 out of the 62 searched traits/anomalies were not found [prevalence = 0%]). Microdontia and accessory cusps on the marginal ridge of the second mandibular molars were significantly more common in women (*P* < 0.05). Canine talon cusp and distal trigonid crest of the second mandibular molars were more prevalent in men (*P* < 0.05). Shoveling, canine talon cusp, canine distal accessory ridge, and accessory cusp in the first premolar might be more prevalent in skeletal Angle class II; whereas, accessory cusp in the mesial marginal ridge of the second premolar might be rather more prevalent in skeletal Angle class I (*P* < 0.05). Few dental traits were positively and moderately or strongly correlated with each other (Spearman Rho ≥ 0.4, *P* < 0.0005).

**Conclusions:**

Sex dimorphism was uncommon in nonmetric dental traits/anomalies. Skeletal malocclusions may be associated with a few dental abnormalities or variations.

**Supplementary Information:**

The online version contains supplementary material available at 10.1186/s12903-022-02481-y.

## Introduction

Nonmetric dental traits are significant to clinical dentistry (e.g., orthodontics, and prosthodontics), dental anatomy/morphology, anthropology, oral pathology, and forensic dentistry [1]. Differences in the shape and size of the teeth are an important factor related to the etiology of malocclusion [2, 3].

The teeth are resistant to decomposition, destructive agents, fire, and time; they can convey information about ethnicity and gender; they also allow assessment of origins, the migratory chain, and habits/diets of populations [4–7]. Among the biological variations existing in populations, nonmetric dental traits are key factors for scientists who search for the link between populations’ biological history and their phenotypes. Dental anthropology has great significance in the study of populations’ variations because of the existence of numerous independent traits, excellent preservation, evolutionary conservatism free of selective pressure, genetic determination, interpopulational variation, and the simple assessment of live individuals such as in fossils [6, 8–11].

Nonmetric characteristics of dental crowns are phenotypic forms of dental enamel that result from the indirect process of secretion of mineral mediators by dental morphogenesis proteins and are expressed by the human genome of each individual. They can be positive (cusp) or negative structures (pit and groove) that can appear in a specific location and to varying degrees [12]. These features are described using different designations. They have a high classification value and can be used for biological prediction between different populations and comparative analysis of the history, culture, and biological progress of early humans and modern humans [12].

Since there was no study or just a few ones on a large set of so many dental anomalies, this study was conducted.

## Materials and methods

This cross-sectional study was performed on 662 maxillary and mandibular dental casts of 331 patients (over 9000 teeth). The patients were selected randomly from Iranian patients attending the Orthodontic Department and two private orthodontic clinics in Ahvaz, Iran. For collecting the data, all the available patients’ records, as well as their archival casts and radiographs, were consecutively evaluated until reaching the desired sample size. A total of 809 patient records (along with their casts and radiographs) were evaluated. The inclusion criteria were being of Iranian descent, 12–35 years old, and having a complete permanent dentition (except the third molars) with no more than 2 extractions. The exclusion criteria were patients with any systemic diseases affecting the teeth or syndromes, cleft lips or palates, any earlier histories of orthodontic, prosthodontic, or surgical treatments. Also excluded were patients who did not have all the permanent teeth completely (except cases of hypodontia, cases of sporadic excluded teeth, cases of one or two extracted teeth, and also except the third molars), or patients with more than two extracted teeth, and a lack of complete eruption of more than two of the existing permanent teeth (including the second molars). The other exclusion criteria were single teeth with visible restorations, caries, crown fractures, or veneers (or a history of them), and teeth that had not been fully erupted. Cases with poor cast quality or a lack of panoramic radiographs or lateral cephalograms in the patient file would be excluded. Information regarding the patients’ age, sex, and type of skeletal malocclusion (Angle classes I, II, and III) was recorded from their files, radiographs, and casts. The data collection was performed from 2018 to 2020. No patient was exposed to X-rays for this research, and all the used radiographs were archival and taken merely for treatment purposes. Protocol ethics were approved by the Research Committee of the University (ethical code: U-98142) under the Helsinki Declaration [13–15].

The sample size was pre-determined as 267 patients using the following formula with conservative parameters: $n=({\mathrm{Z}}^{2}* p*(1- p) )/({\mathrm{d}}^{2})$ where Z = 1.96, *p* (prevalence) = 0.5 (as the most conservative prevalence, i.e., the prevalence yielding the maximum sample size within this formula), and d (precision) = 0.06 (as a conservative precision). The sample size was augmented to 331 patients to ensure greater precision. After obtaining the data, the average prevalence of the traits/anomalies was 20.65%; the highest prevalence was 82.18%. For these average and maximum prevalence rates, sample sizes of 175 and 157 cases would suffice respectively, indicating that the current sample size of 331 cases was adequately large.

### Examinations

**Fig. 1:**
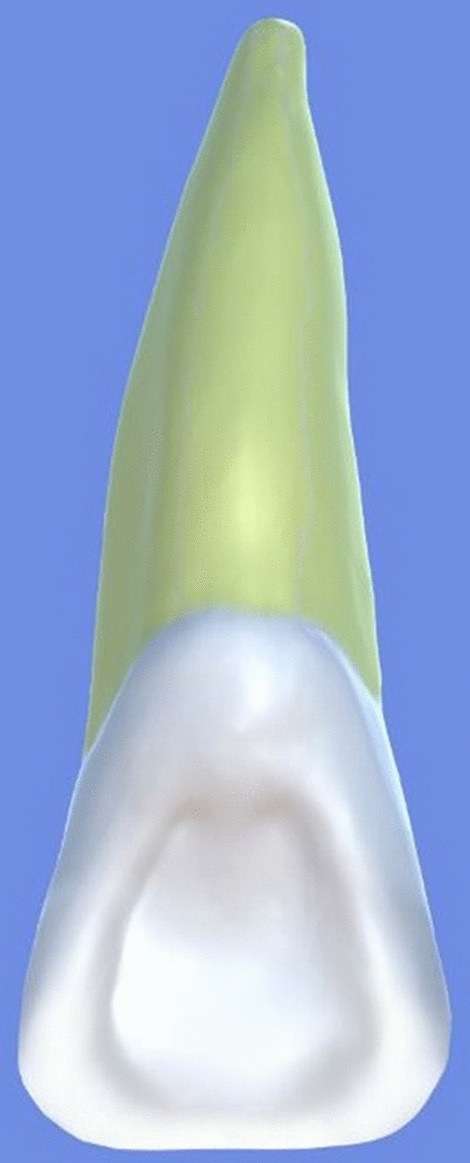
A shovel-shaped incisor. Figures 1 to 13 were created by taking screenshots of an educational Android program with direct permission from the developer and owner (3D Tooth Anatomy 1.0.3; Universal Hospital LP, Richmond, Virginia, USA; developed by Dr Rami Ammoun, assistant professor at Virginia Commonwealth University, Richmond, Virginia, USA) and editing some of the screenshots using 2D image editing software (Photoshop, Adobe, San Jose, California, USA)

**Fig. 2:**
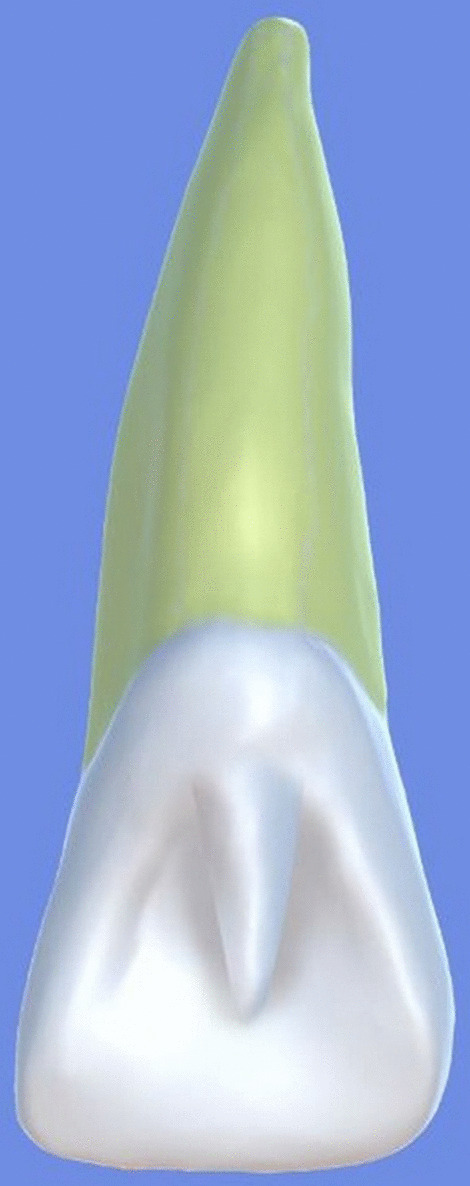
An incisor with a talon cusp

**Fig. 3:**
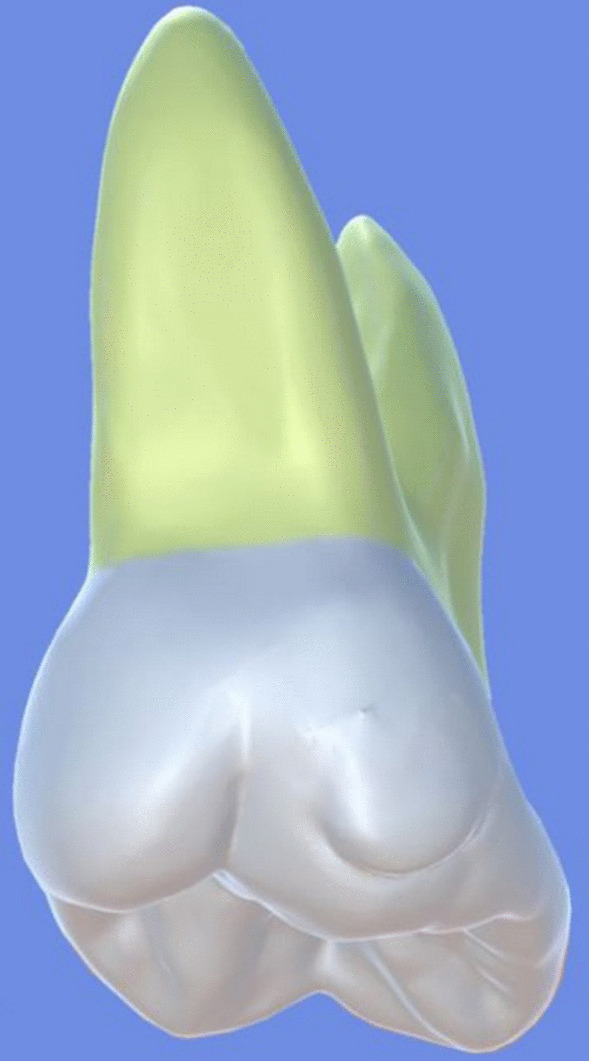
The Carabelli cusp is visible on the palatal surface of the mesiopalatal cusp

**Fig. 4:**
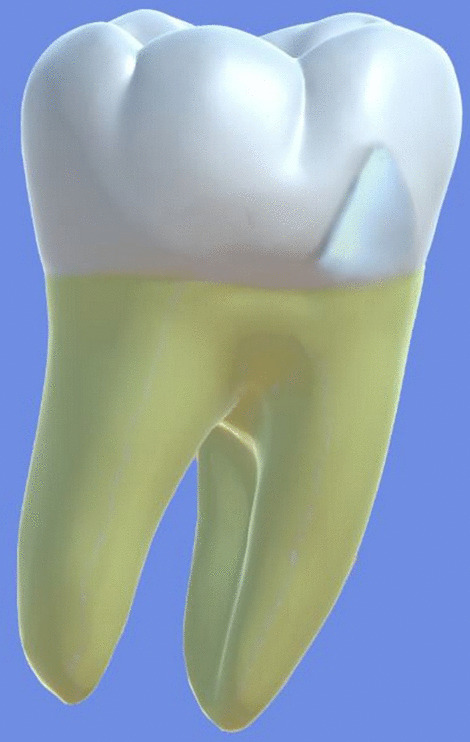
The protostylid trait

**Fig. 5:**
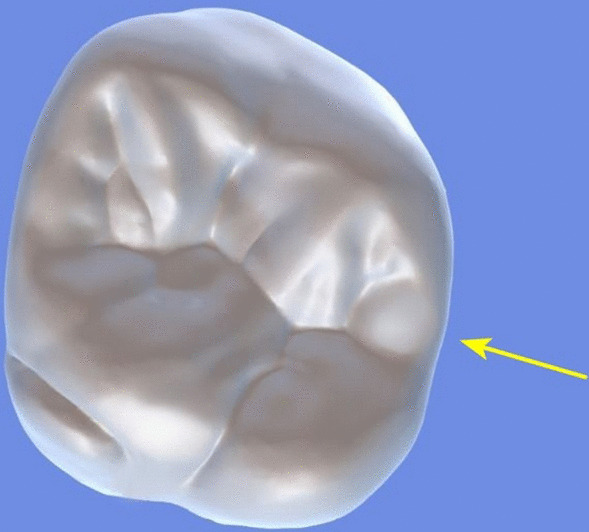
The fifth cusp on the distal side of a maxillary first molar (arrow). The Carabelli cusp is as well visible on the mesiopalatal cusp

**Fig. 6:**
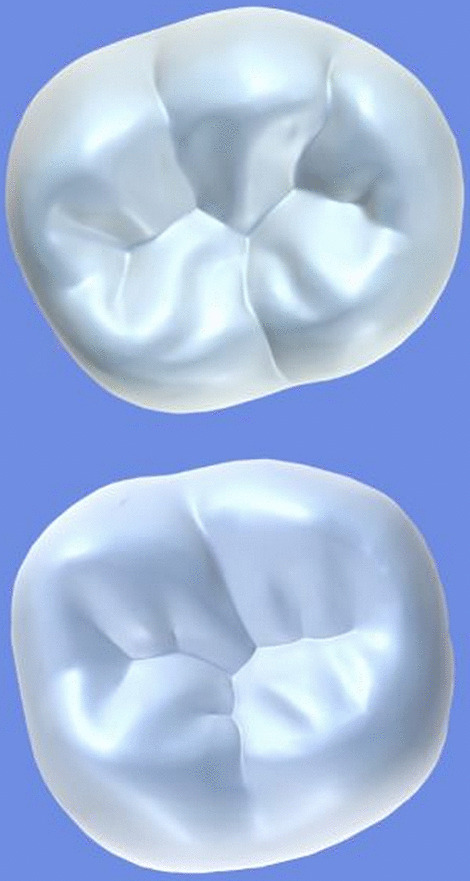
The hypoconulid absence: The upper tooth is a mandibular first molar with all common cusps; the lower one is a mandibular first molar without the hypoconulid cusp

**Fig. 7:**
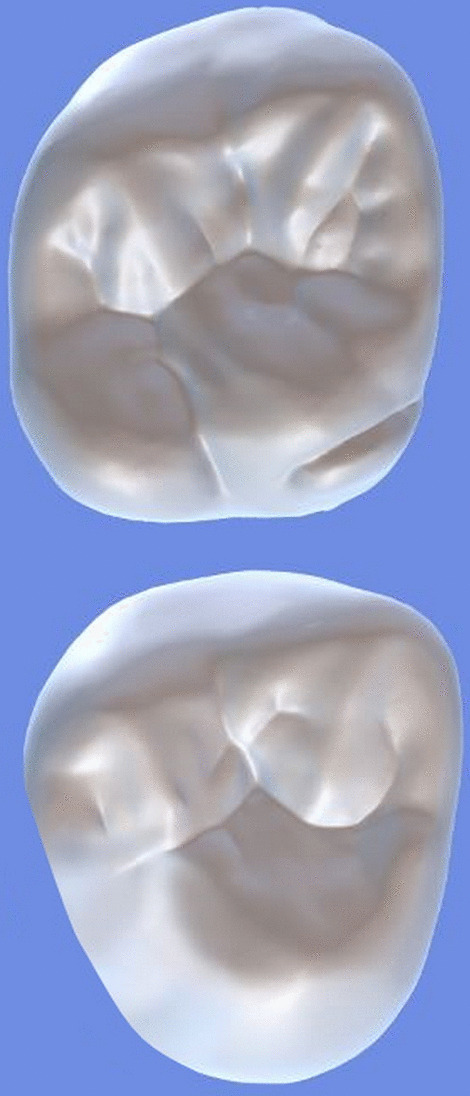
The hypocone absence: The upper image shows a maxillary first molar with 4 cusps; the lower one shows a maxillary second molar without the hypocone cusp

**Fig. 8:**
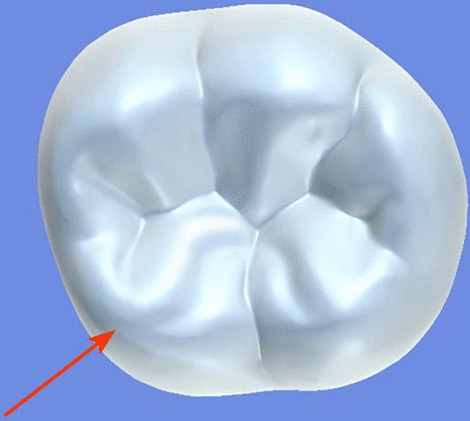
The deflecting wrinkle (arrow) on the mesiolingual occlusal surface of a mandibular molar

**Fig. 9:**
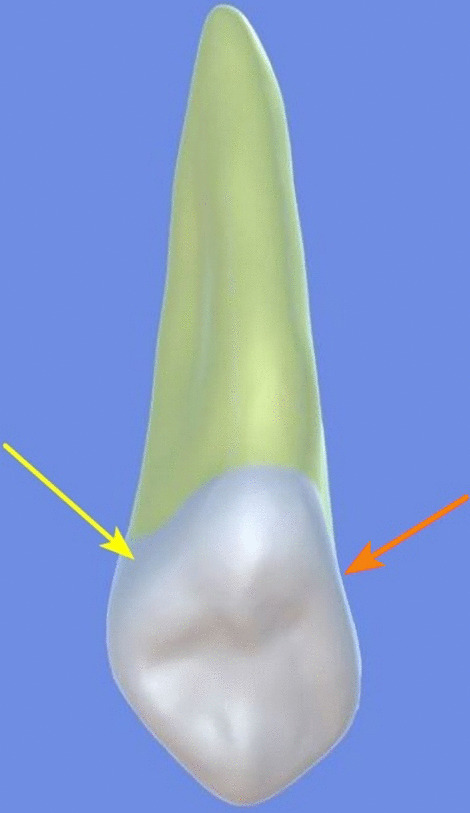
The mesial ridge (yellow arrow) and distal accessory ridge (orange arrow) of the maxillary canine

**Fig. 10:**
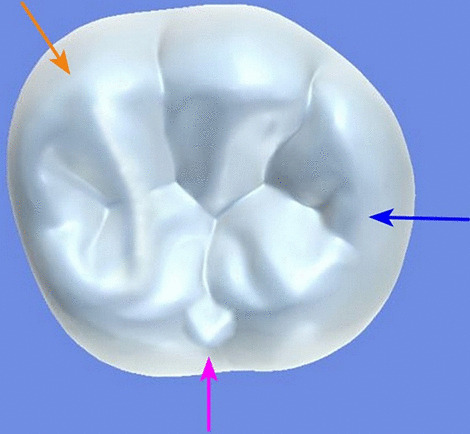
The distal trigonid crest connecting the mesiobuccal and mesiolingual ridges (orange arrow), the sixth cusp on the distal side (blue arrow), and the tuberculum intermedium on the lingual side (purple arrow)

**Fig. 11:**
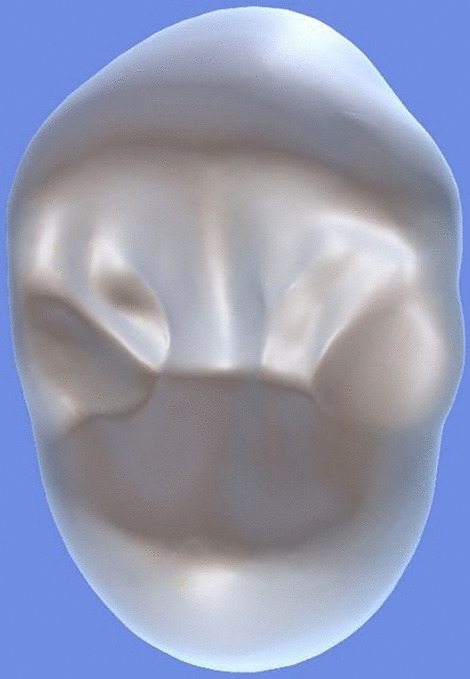
Accessory cusps on the mesial and distal marginal ridges

**Fig. 12:**
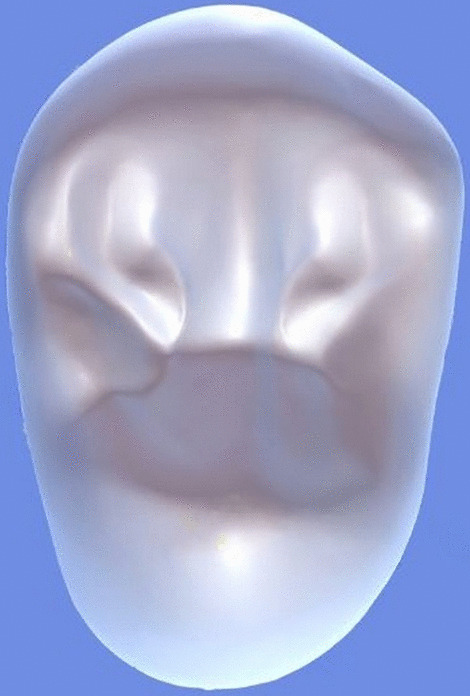
The mesial and distal accessory ridges

**Fig. 13:**
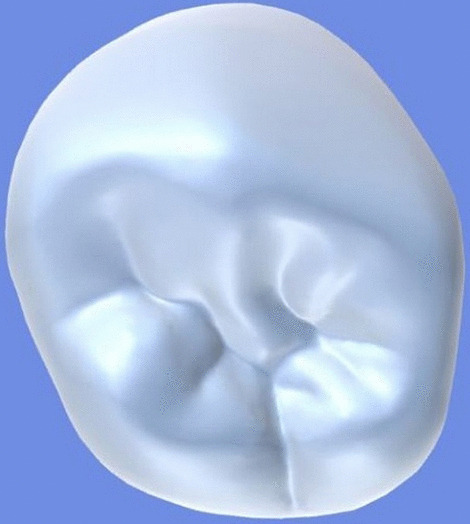
An accessory cusp on the lingual side of a 3-cusp premolar

All archival dental casts had been poured with white dental stone for orthodontic application. All 4 quarters of each patient were examined carefully by two observers (an experienced orthodontist and a trained dentist), twice each. They tried to identify the 60 traits/anomalies mentioned in Table 1 and Figs. 1 to 13 (25 dental traits that might appear in 60 teeth) [6, 16] as well as supernumerary teeth (hyperdontia), microdontia (totaling 62 traits/anomalies), and crowding. Microdontia was defined as noticeably small but normally shaped teeth [16].

**Table 1:** Nonmetric dental traits examined in this study

Maxillary	Definition	Teeth	Mandibular	Definition	Teeth
Shoveling (Fig. 1)	Affected teeth have pronounced lingual marginal ridges and deeper lingual fossae than normal	1, 2, 3	Shoveling (Fig. 1)	Teeth with pronounced lingual marginal ridges and deeper lingual fossae than normal	1, 2, 3
Talon cusp (Fig. 2)	An enamel projection on the cingulum area	1, 2, 3	Talon cusp (Fig. 2)	An enamel projection on the cingulum area	1, 2, 3
Carabelli cusp (Fig. 3)	A non-functional cusp or tubercle on the mesiolingual surface with expression ranges from a slight groove or pit to a large cusp	6, 7	Protostylid (Fig. 4)	A triangular arrangement of cusps with the protostylid cusp located mesiobuccally	6, 7
Fifth cusp (Fig. 5)	An extra enamel projection between the DB and DL cusps	6, 7	Hypoconulid absence (4-cusp) (Fig. 6)	Molars with 4 cusps without the distal cusp (hypoconulid)	6, 7
Hypocone absence (3-cusp) (Fig. 7)	The 2nd molar without the distolingual cusp (hypocone)	7	Deflecting wrinkle (Fig. 8)	The metaconid cusp ridge on the mesiolingual occlusal surface running from the ML cusp toward the central groove and central pit	6, 7
Canine mesial ridge (Fig. 9)	An oblique ridge between the lingual ridge and mesial marginal ridge	3	Distal trigonid crest (Fig. 10)	A transverse ridge that connects two mesial cusps	6, 7
Canine distal accessory ridge (Fig. 9)	An oblique ridge between the lingual ridge and distal marginal ridge (DMR)	3	Tuberculum sextum (sixth cusp) (Fig. 10)	An extra enamel projection on the distal marginal ridge	6, 7
Sixth cusp (tuberculum sextum) (Fig. 10)	An extra enamel projection on the distal marginal ridge	6, 7	Accessory cusp in the mesial marginal ridge (Fig. 11)	An extra enamel projection on the mesial marginal ridge (MMR)	4, 5, 6, 7
Accessory cusp on the mesial marginal ridge (Fig. 11)	An extra enamel projection on the mesial marginal ridge	4, 5, 6, 7	Accessory cusp on the distal marginal ridge (Fig. 11)	An extra enamel projection on the distal marginal ridge	4, 5, 6, 7
Accessory cusp on the distal marginal ridge (Fig. 11)	An extra enamel projection on the distal marginal ridge	4, 5, 6, 7	Mesial accessory ridge (Fig. 12)	The accessory ridge between the MMR and triangular (central) ridge	4, 5
Mesial accessory ridge (Fig. 12)	The accessory ridge between the MMR and triangular (central) ridge	4, 5	Distal accessory ridge (Fig. 12)	The accessory ridge between the DMR and triangular (central) ridge	4, 5
Distal accessory ridge (Fig. 12)	The accessory ridge between the DMR and triangular (central) ridge	4, 5	Accessory cusp on lingual (Fig. 13)	An extra enamel projection on the lingual	4, 5, 7
			Tuberculum intermedium (Fig. 10)	The accessory cusp between the lingual cusps (tuberculum intermedium)	6, 7

These were 25 dental traits/anomalies that could appear in 60 teeth. MMR, mesial marginal ridge; DMR, distal marginal ridge. The used dental notation for numbering the teeth was the alphanumeric notation, in which the numbers 1 to 7 denote the central incisor to the second molar, respectively

Of these 62 traits and anomalies, 44 were found (Table 2); the prevalence of the rest of them was 0%. For hyperdontia diagnosis, also panoramic radiographs were evaluated for possible impacted supernumerary teeth. The data spreadsheets were evaluated by the two observers as well as a third evaluator to find any inconsistencies (which were rather rare). Any cases of inconsistency were re-evaluated on dental casts by both observers. The third evaluator did not check the dental casts.

**Table 2:** The prevalence rates (and Wilson 95% CIs for prevalence rates) of 44 nonmetric traits/anomalies (and crowding) in the sample, men, and women

Trait/abnormality	Percent (n = 331)	Wilson 95% CI		Percent (n = 331)				*P*
				With trait (%)		Without trait (%)		
				Women	Men	Women	Men	
Hyperdontia	0.6	0.2	2.2	0.3	0.3	77.3	22.1	0.347
Microdontia	38.7	33.6	44.0	33.5	5.1	44.1	17.2	**0.002**
Peg-shaped lateral	2.7	1.4	5.1	1.8	0.9	75.8	21.5	0.423
Shoveling on central	52.9	47.5	58.2	41.1	11.8	36.6	10.6	0.974
Shoveling on lateral	55.0	49.6	60.3	42.9	12.1	34.7	10.3	0.855
Shoveling on canine	49.8	44.5	55.2	37.5	12.4	40.2	10.0	0.278
Talon cusp on central	13.3	10.1	17.4	10.0	3.3	67.7	19.0	0.651
Talon cusp on lateral	19.9	16.0	24.6	15.4	4.5	62.2	17.8	0.936
Talon cusp on canine	26.0	21.6	31.0	17.8	8.2	59.8	14.2	**0.019**
Carabelli cusp on U6	60.1	54.8	65.3	45.2	15.2	32.4	7.3	0.147
Carabelli cusp on U7	4.5	2.8	7.3	3.6	0.9	74.0	21.5	0.823
Fifth cusp on U6	1.2	0.5	3.1	1.2	0.0	76.4	22.4	0.280
Hypocone absence—U7	40.8	35.6	46.2	33.8	6.9	43.8	15.4	**0.054**
Tuberculum sextum on L6	2.7	1.4	5.1	1.8	0.9	75.8	21.5	0.423
Hypoconulid absence—L7	82.2	77.7	85.9	65.3	16.9	12.4	5.4	0.097
Protostylid	3.3	1.9	5.9	2.4	0.9	75.2	21.5	0.691
Deflecting wrinkle on L6	24.8	20.4	29.7	19.0	5.7	58.6	16.6	0.838
Deflecting wrinkle on L7	3.0	1.6	5.5	2.4	0.6	75.2	21.8	0.856
Canine mesial ridge	5.7	3.7	8.8	4.5	1.2	73.1	21.1	0.888
Distal trigonid crest on L6	3.6	2.1	6.2	2.7	0.9	74.9	21.5	0.823
Distal trigonid crest on L7	8.2	5.7	11.6	4.8	3.3	72.8	19.0	**0.017**
Canine distal accessory ridge	36.3	31.3	41.6	26.9	9.4	50.8	13.0	0.252
Hypoconulid absence—L6	17.2	13.5	21.7	14.2	3.0	63.4	19.3	0.338
Sixth cusp on U6	0.3	0.1	1.7	0.3	0.0	77.3	22.4	0.591
Fifth cusp on U7	2.1	1.0	4.3	1.5	0.6	76.1	21.8	0.690
Accessory cusp on mesial marginal ridge—4	2.1	1.0	4.3	1.5	0.6	76.1	21.8	0.690
Accessory cusp on mesial marginal ridge—5	2.4	1.2	4.7	1.5	0.9	76.1	21.5	0.298
Accessory cusp on mesial marginal ridge—6	13.6	10.3	17.7	11.2	2.4	66.5	19.9	0.428
Accessory cusp on mesial marginal ridge—7	10.9	8.0	14.7	10.0	0.9	67.7	21.5	**0.032**
Accessory cusp on distal marginal ridge—4	1.8	0.8	3.9	1.2	0.6	76.4	21.8	0.515
Accessory cusp on distal marginal ridge—5	9.7	6.9	13.3	6.9	2.7	70.7	19.6	0.410
Accessory cusp on distal marginal ridge—6	4.2	2.5	7.0	3.6	0.6	74.0	21.8	0.459
Accessory cusp on distal marginal ridge—7	4.2	2.5	7.0	3.6	0.6	74.0	21.8	0.459
Accessory cusp on lingual—L4	23.6	19.3	28.4	17.8	5.7	59.8	16.6	0.627
Accessory cusp on lingual—L5	54.7	49.3	60.0	42.0	12.7	35.6	9.7	0.684
Mesial accessory ridge—4	19.0	15.2	23.6	13.6	5.4	64.0	16.9	0.188
Mesial accessory ridge—5	51.7	46.3	57.0	39.6	12.1	38.1	10.3	0.640
Mesial accessory ridge—6	0.3	0.1	1.7	0.3	0.0	77.3	22.4	0.591
Distal accessory ridge—4	66.5	61.2	71.3	52.6	13.9	25.1	8.5	0.374
Distal accessory ridge—5	73.1	68.1	77.6	55.9	17.2	21.8	5.1	0.389
Tuberculum intermedium on L6	6.0	3.9	9.1	5.1	0.9	72.5	21.5	0.415
Tuberculum intermedium on L7	1.2	0.5	3.1	0.9	0.3	76.7	22.1	0.898
Tuberculum sextum on L7	0.9	0.3	2.6	0.6	0.3	77.0	22.1	0.647
Accessory cusp on lingual—L7	1.5	0.6	3.5	1.2	0.3	76.4	22.1	0.899

The used dental notation for numbering the teeth was the alphanumeric notation, in which the numbers 4 to 7 denote tooth numbers from the first premolar to the second molar, respectively, while the letters U and L indicate the words lower (mandibular) and upper (maxillary), respectively. The associations between traits/anomalies and sex were examined using the chi-square test. Significant *P* values in bold

The results were collected at two levels: (A) at the quarter level (hemi-mandible/hemi-maxilla), which showed each nonmetric trait/anomaly in each quarter; (B) at the patient level, which showed each trait/abnormality plus crowding and Angle classes in individual patients.

### Statistical analyses

Thirty casts were reevaluated by one of the observers about one year after the original assessment, and the interrater and intrarater agreements were calculated to be high or excellent for all the found traits and anomalies (Kappa > 0.6, *P* < 0.05). Descriptive statistics and Wilson 95% confidence intervals (CIs) were calculated for prevalence rates. The ages of men and women were compared using an unpaired *t*-test. Data were also summarized for quarters. Associations between the presence of nonmetric traits besides hyperdontia/microdontia with genders, left/right sides, and skeletal malocclusions (Angle classes I, II, and III) were assessed using a chi-square test. Correlations across dental traits were assessed using a Spearman correlation coefficient. The level of significance was set at 0.05.

## Results

There were 74 men and 257 women in the study. The mean (SD) age of patients was 19.21 ± 4.87 years (range 12–35). Mean ages in men and women were 18.29 ± 20.49 and 18.55 ± 19.76 years, respectively. The sexes were balanced in terms of age (*t*-test, *P* = 0.716). Of the patients, 182 (55.7%), 127 (38.8%), and 18 (5.5%) were skeletal Classes I, II, and III, respectively (the Angle classes of four patients were missing). Crowding was observed in 89 out of 331 cases (26.9%).

Prevalence rates of nonmetric traits/anomalies and their 95% CIs are presented in Table 2. Sexual dimorphism was observed in a few traits: microdontia and accessory cusps on the marginal ridge of the second mandibular molars were significantly more common in women (Table 2). Canine talon cusp and distal trigonid crest of the second mandibular molars were more prevalent in men (Table 2). The prevalence of crowding was 26.9% (CI 22.4–31.9%). It was observed in 22 men and 67 women, without any sex dimorphism (chi-square, *P* = 0.532).

### Associations with skeletal malocclusions

There were significant associations between the skeletal Angle classes with these traits: shoveling of the central and lateral and canine (all the 3 shovelings were rather more frequent in class II), talon cusp on the canine (rather more frequent in class II), canine distal accessory ridge (rather more frequent in class II), accessory cusp in the mesial marginal ridge of the second premolar (mandibular and maxillary combined) (rather more frequent in class I and less frequent in class II), accessory cusp in the first premolar (maxillary and mandibular combined, rather more frequent in class II), and mesial accessory ridge in the first premolar (both maxillary and mandibular, less frequent in class I, Table 3). Crowding was observed in 45, 41, and 2 cases of Classes I, II, and III, respectively with no significant difference across the classes (chi-square, *P* = 0.101).

**Table 3:** Contingency tables show the net frequencies (and %) of 44 nonmetric traits/anomalies and crowding in different skeletal Angle classes, as well as associations between the presence of dental traits and dental occlusion classes

Trait/anomaly	With trait (%)			Without trait (%)			*P*
	Cl I	Cl II	Cl III	Cl I	Cl II	Cl III	
Hyperdontia	1 (50)	1 (50)	0	181 (55.7)	126 (38.8)	18 (5.5)	0.911
Microdontia	66 (52)	51 (40.2)	10 (7.9)	116 (58)	76 (38)	8 (4)	0.257
Peg-shaped lateral	3 (33.3)	6 (66.7)	0	179 (56.3)	121 (38.1)	18 (5.7)	0.204
Shoveling on central	84 (48.6)	80 (46.2)	9 (5.2)	98 (63.6)	47 (30.5)	9 (5.8)	**0.014**
Shoveling on lateral	90 (50)	82 (45.6)	8 (4.4)	92 (62.6)	45 (30.6)	10 (6.8)	**0.021**
Shoveling on canine	78 (47.6)	80 (48.8)	6 (3.7)	104 (63.8)	47 (28.8)	12 (7.4)	**0.001**
Talon cusp on central	24 (55.8)	19 (44.2)	0	158 (55.6)	108 (38)	18 (6.3)	0.213
Talon cusp on lateral	31 (47.7)	31 (47.7)	3 (4.6)	151 (57.6)	96 (36.6)	15 (5.7)	0.262
Talon cusp on canine	38 (45.2)	42 (50)	4 (4.8)	144 (59.3)	85 (35)	14 (5.8)	**0.051**
Carabelli cusp on U6	104 (52.5)	81 (40.9)	13 (6.6)	77 (60.2)	46 (35.9)	5 (3.9)	0.316
Carabelli cusp on U7	73 (54.5)	52 (38.8)	9 (6.7)	109 (56.5)	75 (38.9)	9 (4.7)	0.453
Fifth cusp on U6	6 (66.7)	2 (22.2)	1 (11.1)	176 (55.3)	125 (39.3)	17 (5.3)	0.829
Hypocone absence—U7	73 (54.5)	52 (38.8)	9 (6.7)	109 (56.5)	75 (38.9)	9 (4.7)	0.718
Tuberculum sextum on L6	6 (66.7)	2 (22.2)	1 (11.1)	176 (55.3)	125 (39.3)	17 (5.3)	0.500
Hypoconulid absence—L7	152 (56.3)	100 (37)	18 (6.7)	30 (52.6)	27 (47.4)	0	0.074
Protostylid	7 (63.6)	3 (27.3)	1 (9.1)	175 (55.4)	124 (39.2)	17 (5.4)	0.674
Deflecting wrinkle on L6	47 (58)	30 (37)	4 (4.9)	135 (54.9)	97 (39.4)	14 (5.7)	0.878
Deflecting wrinkle on L7	4 (40)	5 (50)	1 (10)	178 (56.2)	122 (38.5)	17 (5.4)	0.559
Canine mesial ridge	12 (63.2)	7 (36.8)	0	170 (55.2)	120 (39)	18 (5.8)	0.513
Distal trigonid crest on L6	7 (58.3)	5 (41.7)	0	175 (55.6)	122 (38.7)	18 (5.7)	0.695
Distal trigonid crest on L7	14 (53.8)	10 (38.5)	2 (7.7)	168 (55.8)	117 (38.9)	16 (5.3)	0.877
Canine distal accessory ridge	52 (43.7)	61 (51.3)	6 (5)	130 (62.5)	66 (31.7)	12 (5.8)	**0.002**
Hypoconulid absence—L6	31 (54.4)	24 (42.1)	2 (3.5)	151 (55.9)	103 (38.1)	16 (5.9)	0.701
Sixth cusp on U6	1 (100)	0	0	181 (55.5)	127 (39)	18 (5.5)	0.671
Fifth cusp on U7	3 (42.9)	4 (57.1)	0	179 (55.9)	123 (38.4)	18 (5.6)	0.543
Accessory cusp on mesial marginal ridge—4	4 (57.1)	3 (42.9)	0	178 (55.6)	124 (38.8)	18 (5.6)	0.808
Accessory cusp on mesial marginal ridge—5	5 (62.5)	1 (12.5)	2 (25)	177 (55.5)	126 (39.5)	16 (5)	**0.027**
Accessory cusp on mesial marginal ridge—6	28 (62.2)	13 (28.9)	4 (8.9)	154 (54.6)	114 (40.4)	14 (5)	0.244
Accessory cusp on mesial marginal ridge—7	21 (58.3)	15 (41.7)	0	161 (55.3)	112 (38.5)	18 (6.2)	0.307
Accessory cusp on distal marginal ridge—4	3 (50)	2 (33.3)	1 (16.7)	179 (55.8)	125 (38.9)	17 (5.3)	0.480
Accessory cusp on distal marginal ridge—5	18 (56.3)	10 (31.3)	4 (12.5)	164 (55.6)	117 (39.7)	14 (4.7)	0.159
Accessory cusp on distal marginal ridge—6	8 (57.1)	6 (42.9)	0	174 (55.6)	121 (38.7)	18 (5.8)	0.647
Accessory cusp on distal marginal ridge—7	5 (38.5)	8 (61.5)	0	174 (55.4)	122 (38.9)	18 (5.7)	0.660
Accessory cusp on lingual—L4	31 (39.7)	43 (55.1)	4 (5.1)	151 (60.6)	84 (33.7)	14 (5.6)	**0.003**
Accessory cusp on lingual—L5	92 (51.1)	80 (44.4)	8 (4.4)	90 (61.2)	47 (32)	10 (6.8)	0.062
Mesial accessory ridge—4	41 (66.1)	21 (33.9)	0	141 (53.2)	106 (40)	18 (6.8)	**0.045**
Mesial accessory ridge—5	95 (56.2)	67 (39.6)	7 (4.1)	87 (55.1)	60 (38)	11 (7)	0.533
Mesial accessory ridge—6	1 (100)	0	0	181 (55.5)	127 (39)	18 (5.5)	0.671
Distal accessory ridge—4	114 (52.3)	92 (42.2)	12 (5.5)	68 (62.4)	35 (32.1)	6 (5.5)	0.198
Distal accessory ridge—5	130 (54.2)	97 (40.4)	13 (5.4)	52 (59.8)	30 (34.5)	5 (5.7)	0.621
Tuberculum intermedium on L6	15 (75)	5 (25)	0	167 (54.4)	122 (39.7)	18 (5.9)	0.161
Tuberculum intermedium on L7	2 (50)	2 (50)	0	180 (55.7)	125 (38.7)	18 (5.6)	0.829
Tuberculum sextum on L7	0	3 (100)	0	182 (56.2)	124 (38.3)	18 (5.6)	0.092
Accessory cusp on lingual—L7	4 (80)	1 (20)	0	178 (55.3)	126 (39.1)	18 (5.6)	0.526

The alphanumeric notation is used for numbering the teeth. Where the jaw is not specified (by U or L letters), data from both jaws are combined. U, Upper; L, lower; the numbers 4–7 denote tooth numbers from the first premolar to the second molar. The *P* value is calculated using the chi-square test by comparing prevalence rates of the traits/anomalies in different classes. Significant *P* values in bold

### Quarter level analyses

The summary of all cases at the quarter level, in different hemimandibles and hemimaxillae, is presented in Table 4. All dental traits/anomalies were evenly distributed on the right and left sides (all chi-square *P* values > 0.1, Table 4).

**Table 4:** The net (and %) prevalence rates of 44 nonmetric traits/anomalies in different sexes, jaws, and sides

Trait/anomaly	Presence	Female (%)				Male (%)			
		Maxilla		Mandible		Maxilla		Mandible	
		Right	Left	Right	Left	Right	Left	Right	Left
Supernumerary	No	257 (19.4)	257 (19.4)	256 (19.3)	256 (19.3)	73 (5.5)	73 (5.5)	74 (5.6)	74 (5.6)
	Yes	0	0	1 (0.1)	1 (0.1)	1 (0.1)	1 (0.1)	0	0
Microdontia	None	169 (12.8)	170 (12.8)	193 (14.6)	194 (14.7)	57 (4.3)	61 (4.6)	65 (4.9)	66 (5)
	Local	37 (2.8)	36 (2.7)	18 (1.4)	17 (1.3)	9 (0.7)	5 (0.4)	3 (0.2)	2 (0.2)
	General	51 (3.9)	51 (3.9)	46 (3.5)	46 (3.5)	8 (0.6)	8 (0.6)	6 (0.5)	6 (0.5)
Peg-Shaped lateral	No	252 (19)	254 (19.2)	257 (19.4)	257 (19.4)	71 (5.4)	73 (5.5)	74 (5.6)	74 (5.6)
	Yes	5 (0.4)	3 (0.2)	0	0	3 (0.2)	1 (0.1)	0	0
Shoveling on central	No	122 (9.2)	125 (9.4)	253 (19.1)	253 (19.1)	35 (2.6)	40 (3)	72 (5.4)	72 (5.4)
	Yes	135 (10.2)	132 (10)	4 (0.3)	4 (0.3)	39 (2.9)	34 (2.6)	2 (0.2)	2 (0.2)
Shoveling on lateral	No	121 (9.1)	129 (9.7)	239 (18.1)	243 (18.4)	36 (2.7)	37 (2.8)	64 (4.8)	65 (4.9)
	Yes	136 (10.3)	128 (9.7)	18 (1.4)	14 (1.1)	38 (2.9)	37 (2.8)	10 (0.8)	9 (0.7)
Shoveling on canine	No	152 (11.5)	162 (12.2)	172 (13)	175 (13.2)	41 (3.1)	41 (3.1)	45 (3.4)	44 (3.3)
	Yes	105 (7.9)	95 (7.2)	85 (6.4)	82 (6.2)	33 (2.5)	33 (2.5)	29 (2.2)	30 (2.3)
Talon cusp on central	No	229 (17.3)	228 (17.2)	257 (19.4)	257 (19.4)	67 (5.1)	63 (4.8)	74 (5.6)	74 (5.6)
	Yes	28 (2.1)	29 (2.2)	0	0	7 (0.5)	11 (0.8)	0	0
Talon cusp on lateral	No	217 (16.4)	210 (15.9)	257 (19.4)	257 (19.4)	63 (4.8)	60 (4.5)	74 (5.6)	73 (5.5)
	Yes	40 (3)	47 (3.5)	0	0	11 (0.8)	14 (1.1)	0	1 (0.1)
Talon cusp on canine	No	208 (15.7)	206 (15.6)	255 (19.3)	256 (19.3)	51 (3.9)	51 (3.9)	74 (5.6)	74 (5.6)
	Yes	49 (3.7)	51 (3.9)	2 (0.2)	1 (0.1)	23 (1.7)	23 (1.7)	0	0
Carabelli’s cusp—U6	No	120 (9.1)	119 (9)	257 (19.4)	257 (19.4)	26 (2)	27 (2)	74 (5.6)	74 (5.6)
	Yes	136 (10.3)	137 (10.4)	0	0	48 (3.6)	47 (3.6)	0	0
Carabelli’s cusp—U7	No	246 (18.6)	250 (18.9)	257 (19.4)	257 (19.4)	72 (5.4)	71 (5.4)	74 (5.6)	74 (5.6)
	Yes	11 (0.8)	7 (0.5)	0	0	2 (0.2)	3 (0.2)	0	0
Fifth cusp—U6	No	253 (19.1)	255 (19.3)	257 (19.4)	257 (19.4)	74 (5.6)	74 (5.6)	74 (5.6)	74 (5.6)
	Yes	4 (0.3)	2 (0.2)	0	0	0	0	0	0
Hypocone absence (3-cusp U7)	No	151 (11.4)	154 (11.6)	257 (19.4)	257 (19.4)	53 (4)	53 (4)	74 (5.6)	74 (5.6)
	Yes	106 (8)	103 (7.8)	0	0	21 (1.6)	21 (1.6)	0	0
Tuberculum sextum—L6	No	257 (19.4)	257 (19.4)	253 (19.1)	252 (19)	74 (5.6)	74 (5.6)	73 (5.5)	71 (5.4)
	Yes	0	0	4 (0.3)	5 (0.4)	0	0	1 (0.1)	3 (0.2)
Hypoconulid absence (4-cusp L7)	No	257 (19.4)	257 (19.4)	44 (3.3)	44 (3.3)	74 (5.6)	74 (5.6)	20 (1.5)	18 (1.4)
	Yes	0	0	213 (16.1)	213 (16.1)	0	0	54 (4.1)	56 (4.2)
Protostylid	No	257 (19.4)	257 (19.4)	251 (19)	252 (19)	73 (5.5)	74 (5.6)	72 (5.4)	72 (5.4)
	Yes	0	0	6 (0.5)	5 (0.4)	1 (0.1)	0	2 (0.2)	2 (0.2)
Deflecting wrinkle‏ on L6	No	257 (19.4)	257 (19.4)	209 (15.8)	205 (15.5)	74 (5.6)	74 (5.6)	58 (4.4)	60 (4.5)
	Yes	0	0	48 (3.6)	52 (3.9)	0	0	16 (1.2)	14 (1.1)
Deflecting wrinkle‏ on L7	No	257 (19.4)	257 (19.4)	253 (19.1)	250 (18.9)	74 (5.6)	74 (5.6)	74 (5.6)	72 (5.4)
	Yes	0	0	4 (0.3)	7 (0.5)	0	0	0	2 (0.2)
Canine mesial ridge	No	245 (18.5)	249 (18.8)	257 (19.4)	257 (19.4)	70 (5.3)	71 (5.4)	73 (5.5)	73 (5.5)
	Yes	12 (0.9)	8 (0.6)	0	0	4 (0.3)	3 (0.2)	1 (0.1)	1 (0.1)
Distal trigonid crest—L6	No	257 (19.4)	257 (19.4)	250 (18.9)	252 (19)	74 (5.6)	74 (5.6)	71 (5.4)	73 (5.5)
	Yes	0	0	7 (0.5)	5 (0.4)	0	0	3 (0.2)	1 (0.1)
Distal trigonid crest—L7	No	257 (19.4)	257 (19.4)	246 (18.6)	246 (18.6)	74 (5.6)	74 (5.6)	67 (5.1)	66 (5)
	Yes	0	0	11 (0.8)	11 (0.8)	0	0	7 (0.5)	8 (0.6)
Canine distal accessory ridge	No	181 (13.7)	186 (14)	239 (18.1)	241 (18.2)	47 (3.5)	54 (4.1)	55 (4.2)	59 (4.5)
	Yes	76 (5.7)	71 (5.4)	18 (1.4)	16 (1.2)	27 (2)	20 (1.5)	19 (1.4)	15 (1.1)
Hypoconulid absence (4-cusp L6)	No	257 (19.4)	257 (19.4)	213 (16.1)	214 (16.2)	74 (5.6)	74 (5.6)	65 (4.9)	64 (4.8)
	Yes	0	0	44 (3.3)	43 (3.2)	0	0	9 (0.7)	10 (0.8)
Sixth cusp—U6	No	256 (19.3)	256 (19.3)	257 (19.4)	257 (19.4)	74 (5.6)	74 (5.6)	74 (5.6)	74 (5.6)
	Yes	1 (0.1)	1 (0.1)	0	0	0	0	0	0
Fifth cusp—U7	No	253 (19.1)	254 (19.2)	257 (19.4)	257 (19.4)	72 (5.4)	73 (5.5)	74 (5.6)	74 (5.6)
	Yes	4 (0.3)	3 (0.2)	0	0	2 (0.2)	1 (0.1)	0	0
Accessory cusp on mesial marginal ridge—4	No	253 (19.1)	253 (19.1)	257 (19.4)	257 (19.4)	73 (5.5)	73 (5.5)	74 (5.6)	74 (5.6)
	Yes	4 (0.3)	4 (0.3)	0	0	1 (0.1)	1 (0.1)	0	0
Accessory cusp on mesial marginal ridge—5	No	254 (19.2)	255 (19.3)	255 (19.3)	256 (19.3)	73 (5.5)	72 (5.4)	73 (5.5)	74 (5.6)
	Yes	3 (0.2)	2 (0.2)	2 (0.2)	1 (0.1)	1 (0.1)	2 (0.2)	1 (0.1)	0
Accessory cusp on mesial marginal ridge—6	No	231 (17.4)	231 (17.4)	251 (19)	254 (19.2)	67 (5.1)	68 (5.1)	74 (5.6)	74 (5.6)
	Yes	26 (2)	26 (2)	6 (0.5)	3 (0.2)	7 (0.5)	6 (0.5)	0	0
Accessory cusp on mesial marginal ridge—7	No	231 (17.4)	232 (17.5)	254 (19.2)	254 (19.2)	71 (5.4)	72 (5.4)	74 (5.6)	74 (5.6)
	Yes	26 (2)	25 (1.9)	3 (0.2)	3 (0.2)	3 (0.2)	2 (0.2)	0	0
Accessory cusp on distal marginal ridge—4	No	253 (19.1)	254 (19.2)	257 (19.4)	257 (19.4)	72 (5.4)	73 (5.5)	74 (5.6)	74 (5.6)
	Yes	4 (0.3)	3 (0.2)	0	0	2 (0.2)	1 (0.1)	0	0
Accessory cusp on distal marginal ridge—5	No	249 (18.8)	248 (18.7)	248 (18.7)	247 (18.7)	67 (5.1)	68 (5.1)	72 (5.4)	73 (5.5)
	Yes	8 (0.6)	9 (0.7)	9 (0.7)	10 (0.8)	7 (0.5)	6 (0.5)	2 (0.2)	1 (0.1)
Accessory cusp on distal marginal ridge—6	No	254 (19.2)	256 (19.3)	250 (18.9)	249 (18.8)	73 (5.5)	73 (5.5)	73 (5.5)	73 (5.5)
	Yes	3 (0.2)	1 (0.1)	7 (0.5)	8 (0.6)	1 (0.1)	1 (0.1)	1 (0.1)	1 (0.1)
Accessory cusp on distal marginal ridge—7	No	253 (19.1)	250 (18.9)	254 (19.2)	255 (19.3)	74 (5.6)	73 (5.5)	73 (5.5)	73 (5.5)
	Yes	4 (0.3)	7 (0.5)	3 (0.2)	2 (0.2)	0	1 (0.1)	1 (0.1)	1 (0.1)
Accessory cusp on lingual—L4	No	257 (19.4)	257 (19.4)	205 (15.5)	214 (16.2)	74 (5.6)	74 (5.6)	60 (4.5)	58 (4.4)
	Yes	0	0	52 (3.9)	43 (3.2)	0	0	14 (1.1)	16 (1.2)
Accessory cusp on lingual—L5	No	254 (19.2)	256 (19.4)	132 (10)	152 (11.5)	74 (5.6)	74 (5.6)	40 (3)	36 (2.7)
	Yes	0	0	125 (9.5)	105 (8)	0	0	34 (2.6)	38 (2.9)
Mesial accessory ridge—4	No	240 (18.1)	233 (17.6)	241 (18.2)	245 (18.5)	68 (5.1)	66 (5)	66 (5)	68 (5.1)
	Yes	17 (1.3)	24 (1.8)	16 (1.2)	12 (0.9)	6 (0.5)	8 (0.6)	8 (0.6)	6 (0.5)
Mesial accessory ridge—5	No	166 (12.5)	174 (13.1)	224 (16.9)	220 (16.6)	42 (3.2)	47 (3.5)	58 (4.4)	61 (4.6)
	Yes	91 (6.9)	83 (6.3)	33 (2.5)	37 (2.8)	32 (2.4)	27 (2)	16 (1.2)	13 (1)
Mesial accessory ridge—6	No	257 (19.4)	257 (19.4)	256 (19.3)	256 (19.3)	74 (5.6)	74 (5.6)	74 (5.6)	74 (5.6)
	Yes	0	0	1 (0.1)	1 (0.1)	0	0	0	0
Distal accessory ridge—4	No	168 (12.7)	170 (12.8)	137 (10.3)	129 (9.7)	45 (3.4)	55 (4.2)	42 (3.2)	39 (2.9)
	Yes	89 (6.7)	87 (6.6)	120 (9.1)	128 (9.7)	29 (2.2)	19 (1.4)	32 (2.4)	35 (2.6)
Distal accessory ridge—5	No	114 (8.6)	131 (9.9)	151 (11.4)	150 (11.3)	25 (1.9)	30 (2.3)	46 (3.5)	42 (3.2)
	Yes	143 (10.8)	126 (9.5)	106 (8)	107 (8.1)	49 (3.7)	44 (3.3)	28 (2.1)	32 (2.4)
Tuberculum intermedium on L6	No	257 (19.4)	257 (19.4)	246 (18.6)	244 (18.5)	73 (5.5)	73 (5.5)	71 (5.4)	73 (5.5)
	Yes	0	0	11 (0.8)	13 (1)	0	0	3 (0.2)	1 (0.1)
Tuberculum intermedium on L7	No	257 (19.4)	257 (19.4)	256 (19.3)	254 (19.2)	74 (5.6)	74 (5.6)	74 (5.6)	73 (5.5)
	Yes	0	0	1 (0.1)	3 (0.2)	0	0	0	1 (0.1)
Tuberculum sextum on L7	No	257 (19.4)	257 (19.4)	256 (19.3)	255 (19.3)	74 (5.6)	74 (5.6)	74 (5.6)	73 (5.5)
	Yes	0	0	1 (0.1)	2 (0.2)	0	0	0	1 (0.1)
Accessory cusp on lingual—L7	No	257 (19.4)	257 (19.4)	256 (19.3)	253 (19.1)	74 (5.6)	74 (5.6)	73 (5.5)	74 (5.6)
	Yes	0	0	1 (0.1)	4 (0.3)	0	0	1 (0.1)	0

The alphanumeric notation is used for numbering the teeth: U, upper; L, lower. The numbers 4 to 7 represent the first premolar to the second molar, respectively

### Correlations among the dental traits/anomalies

There were numerous significant correlations among the traits/anomalies (Additional file 1). The significant correlations that also had moderate or strong positive effect sizes (Rho ≥ 0.4) comprised: correlations among shoveling of different anterior teeth (Rho ≥ 0.4, *P* < 0.0005) as well as correlations between the canine’s distal accessory ridge and shoveling of the anterior teeth (Rho ≥ 0.4, *P* < 0.0005), between talon cusp on the central incisor and talon cusp on the lateral incisor (Rho ≥ 0.4, *P* < 0.0005), correlation between the tuberculum sextum on the lower first molar and fifth cusp on the upper first molar (Rho ≥ 0.4, *P* < 0.0005), correlations among accessory cusp in first premolars with shoveling of canine and to a lower degree shoveling of the other anterior teeth (Rho ≥ 0.4, *P* < 0.0005), correlation between mesial accessory ridge on the second premolar with distal accessory ridge on the first and second premolars (Rho ≥ 0.4, *P* < 0.0005), and the correlation between distal accessory ridge of the first premolar and distal accessory ridge of the second premolar (Rho ≥ 0.4, *P* < 0.0005, Additional file 1).

## Discussion

Studies on associations between the Angle classes with nonmetric dental traits and anomalies are scarce and limited to very few anomalies. A recent study [17] assessed the association between skeletal classes and five dental anomalies; they exhibited that microdontia was associated with class III malocclusion. We could not find such an association, however, even though in both studies skeletal malocclusions had been examined. Perhaps, factors such as the ethnic background as well as other methodological factors including sample characteristics may be responsible for the differing results. Still, both studies failed to find a link between hyperdontia and Angle classes. More studies are needed in this regard before explaining potential reasons for the disputes or agreements.

Researchers from various branches of anthropology have documented human variations in different populations and have concluded that phenotypic and genotypic characteristics of populations are related to the geographical distance between them [4, 18–21]. Regionalized anthropological research can replace the classical division of humankind (as Caucasians, Negroids, and Mongoloids) as an important path for historical, evolutionary, and forensic studies [22–25]. Skeletal assessments comprise metric and nonmetric methods that can allow for designing a biological profile and identification [6, 26].

The characteristics of nonmetric traits are primarily used to predict human identity, gender, and origin [14], although as confirmed in this study, few nonmetric traits might have sex dimorphism [27]. It seems that the nonmetric properties of tooth crowns rarely have gender differences. The statistical relationship between these features is small. A significant geographical variation is seen in the frequency of these features [12]. These characteristics also play a critical role in racial and legal identification. So far, over 135 dental features have been identified in the human dental system, but few have been studied in global research [14] on dental casts, direct clinical evaluation, radiography, and digital photography [14, 28]. The characteristics of nonmetric traits are easily observed and recorded, so they provide us with information about genetic and ethnical variations that occur, to organize populations according to the group-specific evolution process [12]. It should be noted, however, that certain dental traits can disappear due to tooth wear and caries [7, 14].

In the present study, three cases of sexual dimorphism had higher frequencies in women compared to men. Regarding the Carabelli’s cusp, it had a male incidence (27.1%) significantly higher than the female (12.3%) in Brazilians [6], but in the present study and another research [29], no significant sex dimorphism was observed for this trait. The absence of hypocone was higher in women in Brazilians [6] and samples from Southeast Asia, North America, India, and North Africa [29]; however, it was not present in our study. Traits like shoveling, fifth cusp, and absence of hypoconulid showed no significant sexual dimorphism in Brazilians [6], and in the study of Hanihara [29] and Aguirre et al. [12].

It should be noted that all the subjects in this study were orthodontic patients with malocclusion and not normal people. Therefore, their results might not be generalized to the normal population. Since there was no similar study on so many dental traits in normal populations (or even orthodontic patients) sampled from any countries, we could not compare our results extensively. Future studies are warranted to evaluate these traits in the normal populations of different countries. Another limitation was the inclusion of cases with one or two extracted teeth or some excluded teeth, since such teeth might have had some traits or abnormalities and their exclusion confound the findings. Due to the difficulty in collecting the cases, we were limited to tradeoff between a few extracted or excluded teeth in some patients versus discarding the whole patient and all the available precious information altogether. Hence, we preferred to keep all the other information obtainable from a patient at the cost of introducing some rather subtle noise to the data by the extracted or excluded teeth. Finally, the sample was not equally distributed in terms of sex or the Angle classes. Hence, it may influence the finding in regards of association between dental abnormalities with sexual dimorphism or skeletal malocclusion.

## Conclusions

1. The prevalence rates (and 95% CIs) of 44 nonmetric shape/number/size dental traits/anomalies in the Iranian orthodontic patients were documented: they might range between 0.3% and 73.1%, with similar prevalence rates on the right and left sides.
2. Sex dimorphism was uncommon in nonmetric traits/anomalies. (A) It was shown that microdontia, hypocone absence, and accessory cusps on the marginal ridge of the mandibular second molars might be more prevalent in women. (B) Canine talon cusp and distal trigonid crest of the second mandibular molars might be more prevalent in men.
3. The skeletal malocclusions were associated with certain dental traits/abnormalities: (A) Shoveling of all the anterior teeth, talon cusp on the canine, canine distal accessory ridge, and accessory cusp in the first premolar might be more prevalent in skeletal Angle class II; whereas (B) accessory cusp in the mesial marginal ridge of the second premolar might be rather more prevalent in skeletal class I, and (C) mesial accessory ridge of the first premolar might be less frequent in skeletal class I.
4. The occurrences of a few dental traits/anomalies were positively correlated with each other to a moderate or strong extent.

## Supplementary Information

**Additional file 1:** Spearman correlation coefficients among the evaluated nonmetric dental traits/anomalies as well as sex and crowding.

## Data Availability

The datasets generated and/or analyzed during the current study are not publicly available due to authors’ decision, but are available from the corresponding author on reasonable request.
